# Therapeutic effect of ultra-long-lasting human C-peptide delivery against hyperglycemia-induced neovascularization in diabetic retinopathy

**DOI:** 10.7150/thno.81714

**Published:** 2023-04-17

**Authors:** Chan-Hee Moon, Ah-Jun Lee, Hye-Yoon Jeon, Eun-Bin Kim, Kwon-Soo Ha

**Affiliations:** Department of Molecular and Cellular Biochemistry, Kangwon National University School of Medicine, Chuncheon, Kangwon-do 24341, Korea

**Keywords:** neovascularization, diabetic retinopathy, K9-C-peptide, human C-peptide, long-term delivery

## Abstract

**Rationale:** Neovascularization is a hallmark of the late stages of diabetic retinopathy (DR) leading to blindness. The current anti-DR drugs have clinical disadvantages including short circulation half-lives and the need for frequent intraocular administration. New therapies with long-lasting drug release and minimal side effects are therefore needed. We explored a novel function and mechanism of a proinsulin C-peptide molecule with ultra-long-lasting delivery characteristics for the prevention of retinal neovascularization in proliferative diabetic retinopathy (PDR).

**Methods:** We developed a strategy for ultra-long intraocular delivery of human C-peptide using an intravitreal depot of K9-C-peptide, a human C-peptide conjugated to a thermosensitive biopolymer, and investigated its inhibitory effect on hyperglycemia-induced retinal neovascularization using human retinal endothelial cells (HRECs) and PDR mice.

**Results:** In HRECs, high glucose conditions induced oxidative stress and microvascular permeability, and K9-C-peptide suppressed those effects similarly to unconjugated human C-peptide. A single intravitreal injection of K9-C-peptide in mice resulted in the slow release of human C-peptide that maintained physiological levels of C-peptide in the intraocular space for at least 56 days without inducing retinal cytotoxicity. In PDR mice, intraocular K9-C-peptide attenuated diabetic retinal neovascularization by normalizing hyperglycemia-induced oxidative stress, vascular leakage, and inflammation and restoring blood-retinal barrier function and the balance between pro- and anti-angiogenic factors.

**Conclusions:** K9-C-peptide provides ultra-long-lasting intraocular delivery of human C-peptide as an anti-angiogenic agent to attenuate retinal neovascularization in PDR.

## Introduction

Diabetic retinopathy (DR) is a microvascular complication of chronic hyperglycemia and is the leading cause of blindness in developed countries 1, 2. Clinically, DR progresses from non-proliferative diabetic retinopathy (NPDR) to proliferative diabetic retinopathy (PDR). Early signs of NPDR include pericyte loss, the appearance of microaneurysms, and microvascular leakage, resulting in damage to retinal blood vessels 3. Late-stage PDR is associated with pathological neovascularization, or the abnormal formation of new blood vessels, which contributes to serious complications that often require retinal surgery, such as vitreous re-hemorrhage, retinal scarring, and retinal traction detachment 3, 4. Furthermore, neovascularization in PDR leads to break down of the blood-retinal barrier (BRB) and consequent edematous thickening of the macula known as diabetic macular edema (DME), which contributes to vision loss 5. Therefore, prevention of retinal neovascularization is essential for treating DR.

Human proinsulin C-peptide is a 31 amino-acid peptide that is released from pancreatic β-cells into the blood circulation in equimolar concentrations with insulin 2, 6. C-peptide was recently found to have beneficial effects against diabetic complications 6-9. C-peptide protects against diabetic vascular dysfunction by activating AMP-activated protein kinase α and thus inhibiting reactive oxygen species (ROS)-mediated mitochondrial dysfunction and endothelial apoptosis 10. C-peptide also displayed a beneficial effect on early-stage DR by ameliorating vascular endothelial growth factor (VEGF)-mediated microvascular leakage in the retinas of diabetic mice 9. C-peptide was also shown to inhibit hyperglycemia-induced metastasis and pulmonary fibrosis by suppressing ROS-mediated microvascular permeability in the lungs of diabetic mice 11, 12. It is not known, however, whether C-peptide has beneficial effects on neovascularization in PDR.

Because diabetic neovascularization develops in the late stages of DR, its treatment requires long-term drug supplementation. Anti-DR drugs such as anti-VEGF agents and corticosteroids have beneficial effects on retinal neovascularization; however, they do not produce satisfactory outcomes in all patients and can cause side effects due to their requirement for frequent intravitreal administration to maintain therapeutic intraocular drug levels 13, 14. Repeated intravitreal injections can cause adverse effects including anxiety, pain, infection, and tissue damage 15. Moreover, frequent administrations into the vitreous chamber can lead to treatment-limiting vitreous hemorrhage, retinal detachment, and vision loss. C-peptide has a circulating half-life of only 30 min due to rapid enzymatic degradation and renal clearance, which makes prolonged delivery difficult to achieve 16. To overcome these limitations, a long-acting form of C-peptide was developed using polyethylene glycol (PEG), a common approach for prolonged drug delivery, which extended the half-life of C-peptide to 6-7 days in a diabetic neuropathy setting 17. PEG molecules can cause unwanted side effects, however, including immunogenicity, treatment heterogeneity, and decreased drug activity 18. Therefore, further development of a long-lasting C-peptide delivery system to treat PDR is needed.

Elastin-like polypeptides (ELPs) are thermosensitive biopolymers derived from human tropoelastin, a precursor of elastin, composed of repeats of the amino-acid pentamer 'VPGXG' 19. These polymers enable spatiotemporally controlled release of drugs through reversible thermal-phase behavior and temperature-dependent self-organization. ELPs have distinctive physicochemical properties, including thermosensitive sol-gel transition, and are biodegradable and non-toxic in the body 19. To overcome the clinical limitations due to the short half-life of of C-peptide, we previously generated an ELP-conjugated form of human C-peptide called K9-C-peptide 20; however, the application of C-peptide-conjugated biopolymer for prolonged intraocular C-peptide delivery to treat DR has not yet been reported.

We hypothesized that controlled release of human C-peptide from a K9-C-peptide depot in the vitreous chamber would exert a long-term protective effect against diabetic retinal neovascularization. We found that a single intravitreal injection of K9-C-peptide maintained physiological levels of C-peptide in the intraocular space of mice for at least 56 days without inducing cytotoxicity. This ultra-long intraocular delivery of C-peptide attenuated diabetic retinal neovascularization by inhibiting oxidative stress, vascular permeability, BRB disruption, and inflammation and helping to maintain the balance between pro- and anti-angiogenic factors in the retinas of PDR mice. These results suggest that K9-C-peptide has potential as an anti-angiogenic agent for the treatment of PDR.

## Methods

### Generation of diabetic mice

Six-week-old male C57BL/6 mice were obtained from DBL (EumSeong, Korea) and maintained under pathogen-free conditions in a temperature-controlled room with a 12-h light/dark cycle. Diabetic mice were generated by single daily intraperitoneal injections of streptozotocin (50 mg/kg body weight; MilliporeSigma, Burlington, MA, USA) for 5 consecutive days. Streptozotocin was freshly prepared in 100 mmol/L citrate buffer (pH 4.5) as previously described 12. Mice with fasting blood glucose concentrations ≥19 mmol/L, polyuria, and glucosuria were considered diabetic. Blood glucose levels and body weights were monitored in the mice once every two weeks (n = 9). All animal experiments conformed to the *Guide for the Care and Use of Laboratory Animals* (National Institutes of Health; Bethesda, MD, USA) and were approved by the Institutional Animal Care and Use Ethics Committee of Kangwon National University (approval No.: KW-201204-3).

### Intravitreal injection of K9-C-peptide, K8 polypeptide, and human C-peptide

**Figure 1:**
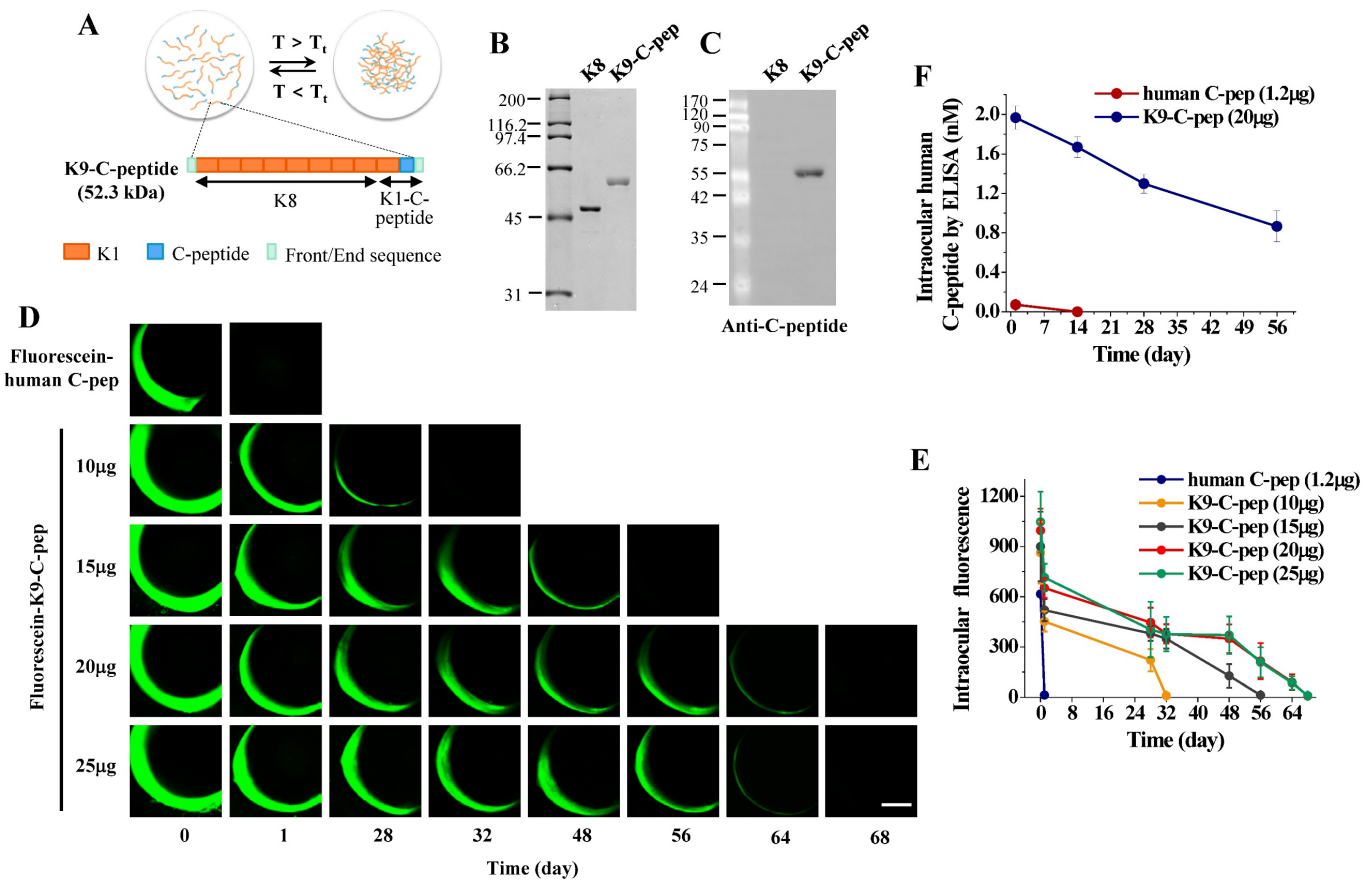
** Pharmacokinetic analysis and intravitreal fluorescence imaging of fluorescein-conjugated K9-C-peptide and human C-peptide in mice. A.** Schematic diagram showing the construction of K9-C-peptide. **B.** Coomassie staining of purified K8 polypeptide (K8) and K9-C-peptide (K9-C-pep). **C.** Western blot analysis of K8 polypeptide and K9-C-peptide using an anti-C-peptide antibody to validate the localization of C-peptide in K9-C-peptide, but not in K8 polypeptide. **D and E.** C57BL/6 mice were intravitreally injected with 1.2 μg fluorescein-conjugated human C-peptide (human C-pep) or the indicated amounts of fluorescein-conjugated K9-C-peptide. Fluorescence images were obtained for 68 days by intravitreal fluorescence imaging. **D.** Representative fluorescence images of fluorescein-conjugated C-peptide and K9-C-peptide in the vitreous chamber. Scale bar, 1,000 μm. **E.** Quantification of fluorescein-conjugated C-peptide and K9-C-peptide levels in mice by measuring fluorescence intensity (n = 6). **F.** C57BL/6 mice were intravitreally injected with equimolar amounts of C-peptide or K9-C-peptide for the indicated times. C-peptide levels in the intraocular space were measured by ELISA (n = 6).

K9-C-peptide, a human C-peptide conjugated to a thermosensitive elastin-like polypeptide, and K8 polypeptide, a negative control for K9-C-peptide, were prepared by inverse transition cycling (ITC) as previously described 21. We generated K9-C-peptide by fusing the gene encoding the K8 polypeptide, which is composed of eight repeats of lysine-containing ELP (ELP-V12K1), to the 5'-end of the gene encoding K1-C-peptide (**Fig. 1A**). The purity and identity of K9-C-peptide and K8 polypeptide were determined by SDS-PAGE and Western blot with a monoclonal antibody against human C-peptide (1:1000, MilliporeSigma).

Six weeks after the first streptozotocin injection, diabetic mice were anesthetized with 3% isoflurane and intravitreally injected with 2 μL phosphate-buffered saline (PBS; vehicle), K9-C-peptide (10 mg/mL; 20 μg), K8 polypeide (8.3 mg/mL; 16.6 μg), or human C-peptide (0.6 mg/mL; 1.2 μg) using a sterile 10 μL syringe (Hamilton, Bonaduz, Switzerland) attached to a 34-gauge needle. The mice were euthanized either 4 weeks after the intravitreal injection for analysis of ROS generation and vascular leakage or 16 weeks after the intravitreal injection for analysis of pathological events related to angiogenesis in the retina.

### Conjugation of K9-C-peptide and C-peptide with NHS-Fluorescein and intravitreal fluorescence imaging

For intravitreal fluorescence imaging, K9-C-peptide and human C-peptide were conjugated with NHS-Fluorescein (Thermo Fisher Scientific, Waltham, MA, USA), as previously reported 21. Briefly, K9-C-peptide (100 μL, 50 mg/mL) or C-peptide (100 μL, 1 mg/mL) in 100 mmol/L sodium bicarbonate buffer (pH 8.3) was mixed with 45 μl or 16 μL, respectively, of 10 mg/ml NHS-Fluorescein in 10% dimethyl sulfoxide and incubated for 2 h on ice. To quench the reaction, 7 μL of 1 mol/L Tris-HCl (pH 8.0) was added to the reaction solution. The reaction mixtures were loaded onto 1.5 mL Sephadex G-25 columns, and fluorescein-conjugated K9-C-peptide and C-peptide were eluted by centrifugation for 3 min at 1,050 × *g*. The levels of conjugated K9-C-peptide and C-peptide levels in the elutes were determined using a human C-peptide ELISA kit (MilliporeSigma).

To analyze the distribution of the polypeptides *in vivo*, mice were anesthetized with 3% isoflurane and intravitreally injected with a total volume of 2 μL containing 0.6 mg/mL (1.2 μg) fluorescein-conjugated C-peptide or 5.0 mg/ml (10 μg), 7.5 mg/mL (15 μg), 10.0 mg/mL (20 μg), or 12.5 mg/mL (25 μg) fluorescein-conjugated K9-C-peptide. Intravitreal fluorescence imaging was performed using confocal microscopy (K1-fluo; Nanoscope Systems, Daejon, Korea). Polypeptide levels were quantitatively analyzed by measuring fluorescence (n = 6).

### Measurement of intraocular C-peptide levels in mice

To analyze the levels of C-peptide released from intravitreal K9-C-peptide depots, mice were anesthetized with 3% isoflurane and intravitreally injected with a total volume of 2 μL containing 0.6 mg/mL (1.2 μg) C-peptide or 10 mg/mL (20 μg) K9-C-peptide. Proteins were extracted from whole eyeballs using lysis buffer containing 50 mmol/L Tris (pH 7.5), 150 mmol/L sodium chloride, 1% Triton X-100, protease inhibitor cocktail (Roche, Basel, Switzerland), 25 mmol/L β-glycerophosphate, and 2 mmol/L sodium orthovanadate. The lysates were centrifuged at 14,000 rpm for 15 min, and then the supernatants were centrifuged at 8,000 rpm for 10 min using Amicon^®^ Ultra 4 mL centrifugal filters with a molecular weight cutoff of 50 kDa (MilliporeSigma). C-peptide levels in the filtered supernatants (n = 6) were measured using a human C-peptide ELISA kit (MilliporeSigma) according to the manufacturer's protocol.

### Measurement of neuro-apoptosis in mouse retinal sections

Neuro-apoptosis in mouse retinas was assessed using an APO-BrdU TUNEL assay kit (BD Bioscience, San Jose, CA, USA) as previously described 22. Briefly, retinal sections (10 μm) were fixed and incubated for 1 h at 37°C with a DNA-labeling solution containing terminal deoxynucleotidyl transferase and 5-bromo-2-deoxyuridine. The sections were then incubated with FITC-labeled 5-bromo-2-deoxyuridine antibody for 30 min and then with 1 μg/mL DAPI (MilliporeSigma) in PBS at room temperature for 10 min. The stained retinal sections were observed by confocal microscopy (K1-Fluo).

### Measurement of IL-6 and VEGF levels in mouse retinas

Lysates were obtained from mouse retinas and centrifuged at 14,000 rpm for 15 min at 4°C. IL-6 and VEGF levels (n = 3, two retinas /experiments) in the lysates were measured using a mouse IL-6 ELISA kit (R&D systems, Minneapolis, MN, USA) and a mouse VEGF ELISA kit (R&D Systems), respectively. ELISA signals were determined using a microplate spectrophotometer (Epoch; BioTek, Winooski, VT, USA).

### H&E staining in mouse retinal sections

Retinal sections (10 μm) were obtained at a distance of about 0.5-0.7 mm from the optic nerve head and fixed with 4% paraformaldehyde for 30 min. H&E staining was performed with Harries hematoxylin solution (MilliporeSigma) for 10 min, followed by 0.2% eosin (MilliporeSigma) for 2 min. The stained sections were observed by confocal microscopy.

### Cell culture

HRECs were purchased from the Applied Cell Biology Research Institute (Cell Systems, Kirkland, WA, USA) and authenticated by short tandem repeat profiling. Cells from passages 7 to 9 were used in experiments. Cells were grown on 2% gelatin-coated plates in M199 medium supplemented with 20% FBS, 3 ng/mL bFGF, 100 U/mL penicillin, and 100 mg/μL streptomycin in a humidified 5% CO_2_ incubator. For experiments, cells were incubated for 12 h in low-serum medium supplemented with 2% FBS, 0.1 ng/mL bFGF, and antibiotics and then treated for 3 days with 5.5 mmol/L D-glucose (normal glucose) or 30 mmol/L D-glucose (high glucose).

### Measurement of ROS generation and permeability assay in HRECs

Intracellular ROS generation was measured in HRECs using CellROX^ TM^ green reagent and dichlorodihydrofluorescein diacetate (H_2_DCFDA; Thermo Fisher Scientific) as previously described 23. Briefly, cells were incubated with either 10 μmol/L H_2_DCFDA for 10 min or 5 μmol/L CellROX^ TM^ green reagent for 30 min in low-serum medium (phenol red-free). Intracellular ROS generation was analyzed by confocal microscopy (n = 6).

*In vitro* endothelial cell monolayer permeability was assessed as previously described 24. Briefly, HRECs were grown to confluence on gelatin-coated inserts of Transwell Permeable Supports (0.4 μm, Costar, Corning, NY, USA) and incubated at 37°C for 3 days in normal or high glucose conditions. The cells were then probed with 1 mg/mL 40-kDa FITC-dextran for 60 min, and the amount of FITC-dextran in the lower chambers (n = 6) was measured by confocal microscopy using well-type arrays 12.

### Measurement of ROS generation in mouse retinal sections

ROS levels in mouse retinas were measured using CellROX^TM^ green reagent as previously described 21. Briefly, mouse retinas were dissected and embedded in Optimal Cutting Temperature compound (Sakura Finetek, Torrance, CA, USA). Unfixed 10 μm retinal cryosections were prepared using a microtome-cryostat and incubated at 37°C with 5 μmol/L CellROX^ TM^ green reagent in serum-free M199 medium for 30 min. The stained retinas were then visualized by confocal microscopy, and ROS levels were quantitatively analyzed by measuring fluorescence (n = 6).

### Measurement of *in vivo* TGase activity in mouse retinas

*In vivo* TGase transamidating activity in whole-mounted retinas was determined by confocal microscopy as previously described 25. Briefly, mice were deeply anesthetized and 48 μL of 100 mmol/L 5-(biotinamido) pentylamine was injected into the left ventricle. Retinas in the Maltese cross configuration were permeabilized with 0.2% Triton X-100 for 30 min and incubated with FITC-conjugated streptavidin (1:200, v/v) for 1 h. The stained retinas were observed by confocal microscopy (K1-fluo), and *in vivo* TGase activity was quantitated by measuring fluorescence intensities in three fields per eye (n = 6).

### Measurement of vascular leakage in mouse retinas

Microvascular leakage in whole-mounted retinas was determined by fluorescein angiography as previously described 26. Briefly, mice were anesthetized, and 1.25 mg 500-kDa FITC-dextran (MilliporeSigma) was injected into the left ventricle and circulated for 5 min. Enucleated eyes were fixed, and whole-mounted retinas were observed by confocal microscopy. Vascular leakage in the vessels of the superficial layer was analyzed by measuring the fluorescence intensity of FITC-dextran extravasated from the retinal vessels (n = 6).

### Western blot analysis

Western blot analysis was performed as previously described 22. Briefly, protein extracts from retinas obtained from each group of mice (n = 6) were resolved by SDS-PAGE and transferred to polyvinylidene fluoride membranes. The membranes were incubated with monoclonal antibodies against pigment epithelium-derived factor (PEDF) (1:2000; Abcam, Cambridge, UK) and IL-1β (1:2000; Santa Cruz Biotechnology; Dallas, TX, USA), followed by incubation with horseradish peroxidase-conjugated secondary antibody. Protein bands were visualized using a ChemiDoc (Bio-Rad, Hercules, CA, USA).

### Immunofluorescence in mouse retinal sections

Expression of VEGF, PEDF, IL-1β, and ionized calcium-binding adaptor molecule 1 (Iba-1) was visualized by immunofluorescence in retinal sections from normal, diabetic (DM), DM+K9-C-peptide, DM+K8 polypeptide, and DM+C-peptide mice. After fixation and permeabilization, retinal sections were incubated overnight with a monoclonal antibody against Iba-1 (1:200; Cell Signaling Technology, Danvers, MA, USA) and polyclonal antibodies against VEGF, PEDF (1:200; Abcam), and IL-1β (1:200; Cell Signaling Technology). The sections were probed for 2 h with FITC-conjugated goat anti-rabbit IgG (1:200; Invitrogen, Carlsbad, CA, USA) and stained for 1 h with Alexa 647-isolectin B4 (IB4) (1:500; Thermo Fisher Scientific). Protein expression was visualized by confocal microscopy and quantitatively analyzed by measuring fluorescence (n = 6).

### Visualization of VE-cadherin and actin filaments in whole-mounted mouse retinas

VE-cadherin in whole-mounted retinas was visualized as previously described 24. Briefly, enucleated eyes were fixed with 4% paraformaldehyde for 45 min at room temperature and then with acetone for 3 min at -20°C. The fixed retinas were permeabilized for 4 h with 1.0% Triton X-100 in PBS and incubated with a monoclonal VE-cadherin antibody (1:200; BD Pharmingen, San Diego, CA, USA). The retinas were then probed with Alexa 647-conjugated goat anti-rat IgG (1:300; Invitrogen), and VE-cadherin in the superficial vessels of the retinas was visualized by confocal microscopy.

Actin filaments in whole-mounted retinas were determined as previously described 24. Briefly, enucleated eyeballs were fixed with 4% paraformaldehyde overnight at 4°C. Following dissection in a tri-lobe configuration, the retinas were permeabilized with 1.0% Triton X-100 in PBS for 4 h and incubated with Alexa Fluor 488 phalloidin (1:200; Molecular Probes, Eugene, OR) for 2 h at room temperature. The superficial vessels in the stained whole-mounted retinas were visualized by confocal microscopy.

### Visualization of microglial activation in whole-mounted mouse retinas

Enucleated eyeballs were fixed with 4% paraformaldehyde for 1 h at room temperature. Retinas were dissected into a tri-lobe configuration and fixed with acetone for 3 min at -20℃. After washing with PBS, the retains were permeabilized in 1.0% Triton X-100 in PBS for 4 h at room temperature and then blocked with 2% BSA in TBS-T overnight at 4℃. The retinas were then incubated overnight at 4℃ with a monoclonal antibody against Iba-1 (1:200; Cell Signaling Technology) and stained with FITC-conjugated goat anti-rabbit IgG (1:200; Invitrogen) for 2 h. Astrocytes in whole-mounted retinas were visualized by confocal microscopy and quantitatively analyzed by measuring fluorescence and the number of microglia (n = 6).

### Visualization and quantitation of angiogenesis in whole-mounted mouse retinas

To visualize angiogenesis, retinas dissected in a tri-lobe configuration were permeabilized for 4 h, blocked overnight at 4℃, and stained with Alexa 647-IB4 (1:500; Thermo Fisher Scientific) for 1 h. The vasculature in whole-mounted retinas was visualized by confocal imaging (F1-flou) using a stitching scan in a 5×5 grid for a total of 25 images per retina. To quantitatively analyze angiogenesis, we measured the area and length of vessels, the number of junctions between vessels, and the lacunarity of the deep plexus layer in a total of nine images from three lobes per retina using the AngioTool software (version 0.6a) (n = 6).

### Statistical analysis

Data were analyzed using OriginPro 2015 software (OriginLab, Northampton, MA, USA). Data are expressed as means ± standard deviation (SD) of three, six, or nine independent experiments. Statistical significance was determined using one-way ANOVA with Holm-Sidak`s multiple comparisons tests. *P* values < 0.05 were considered statistically significant.

## Results

### Characterization and pharmacokinetic analysis of K9-C-peptide in mouse eyes by intravitreal fluorescence imaging and ELISA

K9-C-peptide and K8 polypeptide, a negative control for K9-C-peptide, were expressed in *E. coli*, purified by ITC, and characterized by SDS-PAGE and Western blot (**Fig. 1A and B**). K9-C-peptide and K8 polypeptide appeared as 60-kDa and 50-kDa bands, respectively. Western blot analysis demonstrated the localization of human C-peptide in K9-C-peptide, but not in K8 polypeptide **(Fig. 1C)**. These results indicate that K9-C-peptide was successfully purified, and human C-peptide was localized within the K9-C-peptide.

For pharmacokinetic analysis of K9-C-peptide in mice, we intravitreally injected various amounts of fluorescein-conjugated K9-C-peptide (fluorescein-K9-C-peptide) or 1.2 μg fluorescein-conjugated human C-peptide (fluorescein-C-peptide), an equimolar amount of 20 μg K9-C-peptide, into C57BL/6 mice and performed intravitreal fluorescence imaging over the next 68 days (**Fig. 1D**). The intravitreal fluorescence in mice injected with 10 μg, 15 μg, and 20 μg fluorescein-K9-C-peptide slowly decreased until 28 days, 48 days, and 56 days, respectively, with a weak signal remaining at 64 days after the 20 μg injection; however, the retention time was not extended with the injection of 25 μg fluorescein-K9-C-peptide (**Fig. 1E**). In contrast, the intravitreal fluorescence in mice injected with fluorescein-C-peptide was undetectable at 1 day after the injection. These results demonstrate that human C-peptide was slowly released for at least 56 days from an intravitreal depot of 20 μg K9-C-peptide.

To further study the prolonged release of human C-peptide from an intravitreal K9-C-peptide depot, we intravitreally injected equimolar amounts of K9-C-peptide (20 μg) and human C-peptide (1.2 μg) into the vitreous chamber and monitored intraocular C-peptide levels by ELISA over 56 days (**Fig. 1F**). Consistent with the intravitreal fluorescence imaging results, the intraocular human C-peptide level in mice injected with K9-C-peptide was 1.97 nmol/L at 1 day after injection and then slowly decreased to 0.87 nmol/L at 56 days after injection, which is within the physiological range. In contrast, the intraocular human C-peptide level in mice injected with C-peptide was 0.07 nmol/L at 1 day after injection, suggesting that unbound C-peptide was rapidly cleared from the vitreous chamber. Thus, a single intravitreal injection of K9-C-peptide was sufficient to maintain physiological C-peptide levels for at least 56 days in the intraocular space.

### Intravitreal K9-C-peptide has no cytotoxicity and attenuates hyperglycemia-induced neovascularization in the retinas of PDR mice

**Figure 2:**
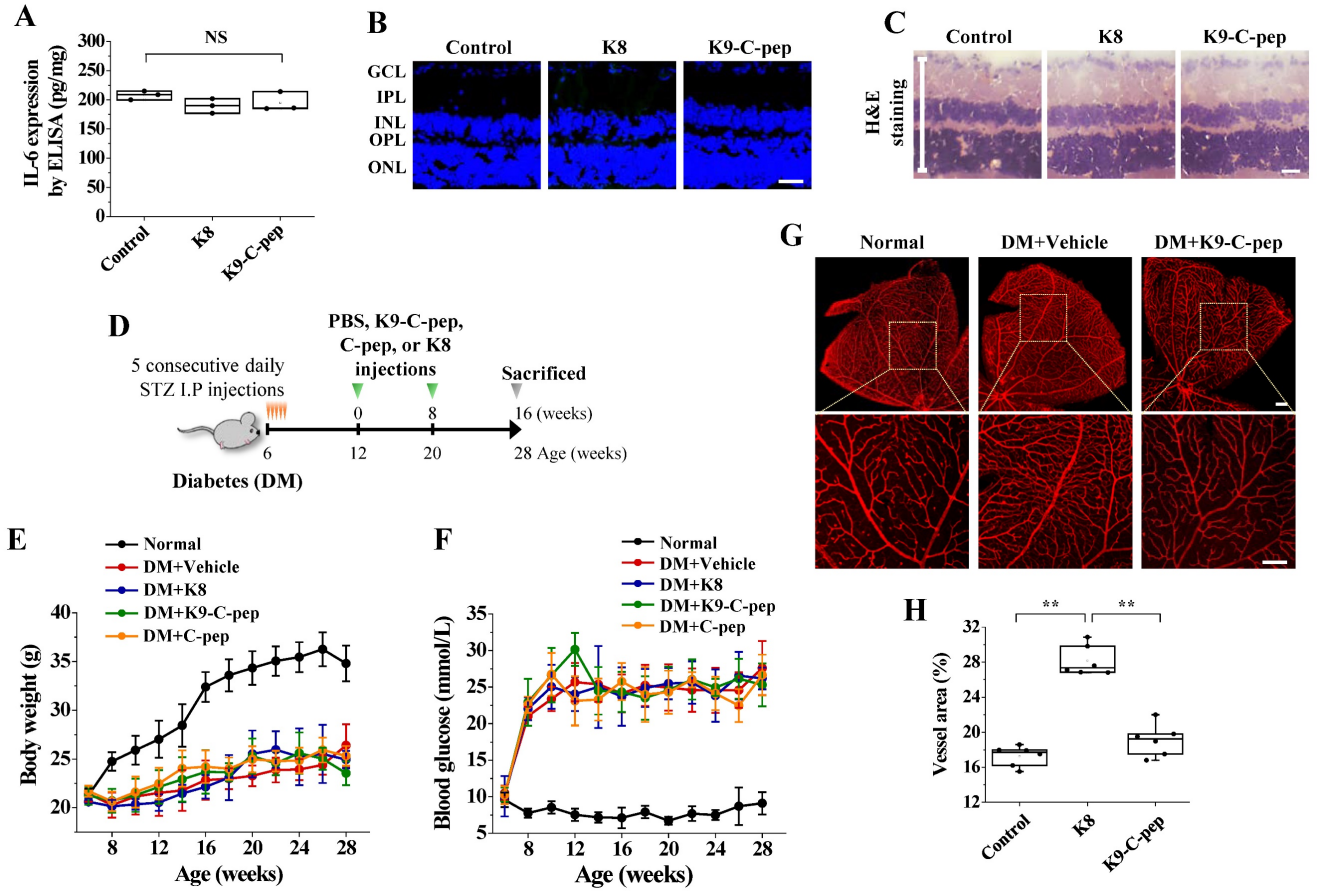
** K9-C-peptide was not cytotoxic in the retina and had a prolonged inhibitory effect against retinal angiogenesis in PDR mice. A-C.** K8 polypeptide (K8) and K9-C-peptide (K9-C-pep) were not cytotoxic in mouse retinas. Eight weeks after intravitreal injection of C57BL/6 mice with K9-C-peptide or K8 polypeptide, the negative control for K9-C-peptide, ocular IL-6 levels, retinal cell apoptosis, and histological changes were analyzed.** A.** Ocular IL-6 levels determined by ELISA of retinal lysates (n = 3). **B.** Retinal apoptosis. Representative images of TUNEL-positive cells (green) with nuclear counterstaining using DAPI (blue) in retinal sections are shown. Scale bar, 100 μm. **C.** No histopathological change was apparent in retinal sections by H&E staining. Scale bar, 100 μm. **D-H.** Six weeks after STZ injection, diabetic mice (DM) were given two intravitreal injections of PBS (vehicle), K9-C-peptide, K8 polypeptide, or human C-peptide (C-pep) over 16 weeks. Angiogenesis was then analyzed in whole-mounted retinas. **D.** Scheme for supplementing diabetic mice by two intravitreal injections of PBS, K9-C-peptide, K8 polypeptide, or C-peptide over 16 weeks. **E and F.** Body weight **(E)** and blood glucose levels** (F)** were monitored every 2 weeks (n = 9). **G and H.** Vascular organization was visualized by Alexa 647-isolectin B4 staining **(G)** and quantified by measuring the vessel percentage area (n = 6) in whole-mounted retinas **(H)**. The bottom row of each group displays enlarged images of the square areas in the top row. Scale bar, 100 μm. Statistical significance was determined using one-way ANOVA with Holm-Sidak`s multiple comparisons test. GCL, ganglion cell layer; IPL, inner plexiform layer; INL, inner nuclear layer; OPL, outer plexiform layer; ONL, outer nuclear layer; NS, non-significant; ^∗∗^* P* < 0.01.

We studied the cytotoxicity of K9-C-peptide 56 days after intravitreal injection in the retinas of C57BL/6 mice using IL-6 ELISA, TUNEL assay, and H&E staining. Neither K9-C-peptide nor K8 peptide induced inflammation, apoptosis, or pathological changes in the retinas **(Fig. 2A-C)**. These results suggest that intravitreal K9-C-peptide is biocompatible and suitable for investigating long-term therapeutic effects on PDR.

To determine if K9-C-peptide has a long-lasting inhibitory effect on diabetic neovascularization in the retinas of PDR mice, we intravitreally injected diabetic mice twice over 16 weeks with K9-C-peptide **(Fig. 2D)**. Compared with non-diabetic mice, diabetic mice exhibited elevated water and food consumption, loss of body weight, and hyperglycemia, and administration of K9-C-peptide had no significant effect on these parameters (**Fig. 2E and F**). The vessel area in the retinas of diabetic mice was increased compared with that in the retinas of non-diabetic mice, and this increase was inhibited by K9-C-peptide (**Fig. 2G and H**). These results suggest that ultra-long delivery of human C-peptide using K9-C-peptide attenuates diabetic neovascularization in the retinas of PDR mice.

### K9-C-peptide inhibits hyperglycemia-induced ROS generation, TGase activation, and microvascular permeability in human retinal endothelial cells (HRECs) and retinas of NPDR mice

**Figure 3:**
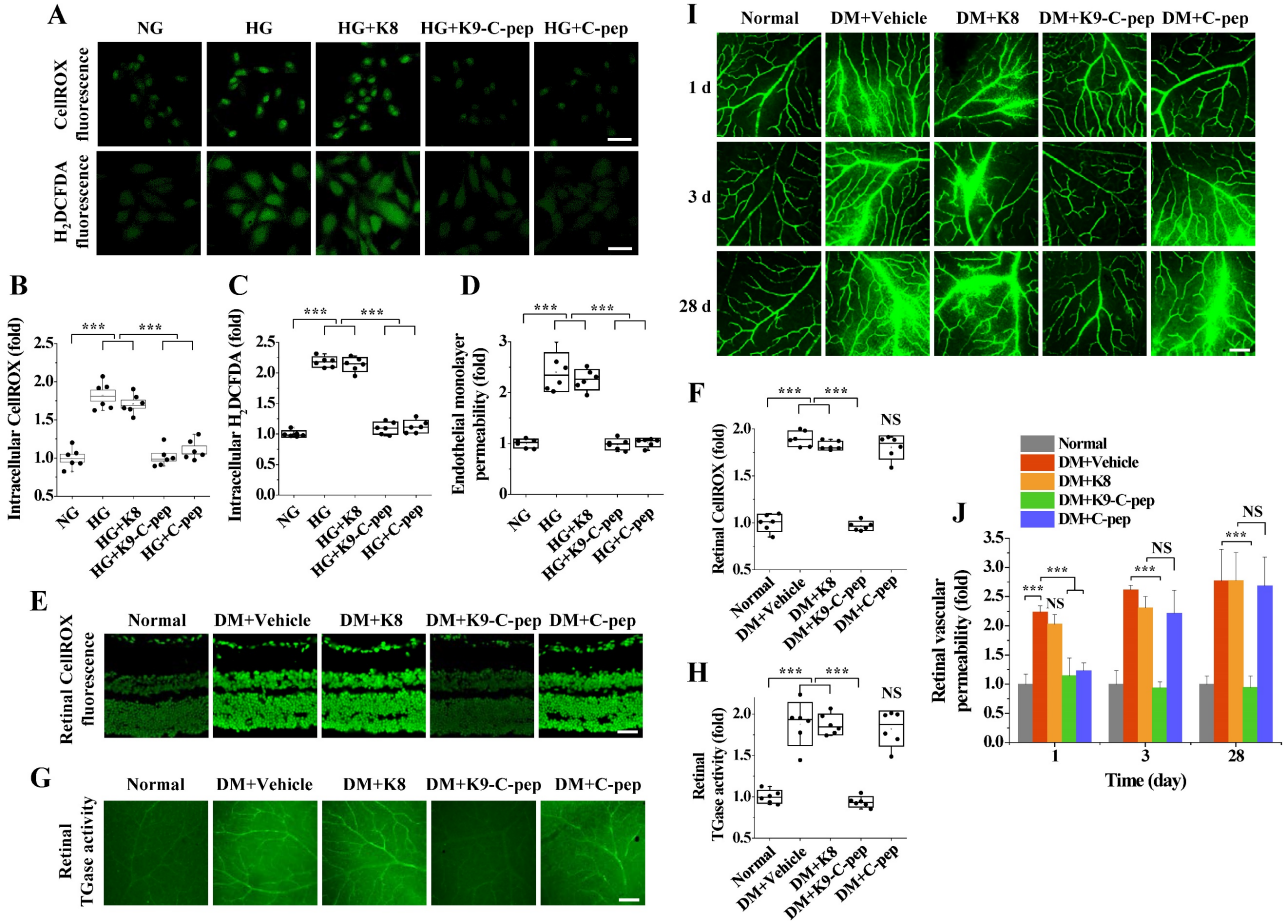
** K9-C-peptide inhibits hyperglycemia-induced ROS generation, TGase activation, and vascular leakage in HRECs and retinas of NPDR mice. A-D.** HRECs were treated for 3 days with normal glucose (NG), high glucose (HG), or high glucose in the presence of 1 nmol/L K8 polypeptide (HG+K8), K9-C-peptide (HG+K9-C-pep), or C-peptide (HG+C-pep). Intracellular ROS generation was visualized by confocal microscopy using CellROX^TM^ green and H_2_DCFDA. **A:** Representative images of ROS generation. Scale bar, 50 μm. **B-C.** ROS generation was quantified by measuring the fluorescence intensities of CellROX^TM^ (B) and H_2_DCFDA (C) (n = 6). **D.** *In vitro* endothelial permeability was determined by 40-kDa FITC-dextran passage (n = 6). E-J. Six weeks after STZ injection, diabetic mice (DM) were supplemented for 4 weeks by a single intravitreal injection of PBS (DM+Vehicle), K8 polypeptide (DM+K8), K9-C-peptide (DM+K9-C-pep), or human C-peptide (DM+C-pep). **E and F.** Retinal ROS levels were visualized by CellROX^TM^ green staining **(E)** and quantified (n = 6) by measuring fluorescence intensity in retinal sections **(F).** Scale bar, 100 μm. **G and H.** TGase activity was visualized in whole-mounted retinas **(G)** and quantified (n = 6) by measuring fluorescence intensity **(F)**. **I and J.** Vascular leakage was visualized using FITC-dextran in whole-mounted retinas **(I)** and quantitatively analyzed (n = 6) by measuring fluorescence intensity **(J)**. Scale bar, 100 μm. Statistical significance was determined using one-way ANOVA with Holm-Sidak`s multiple comparisons test. NS, non-significant; ∗∗∗*P* < 0.001.

We investigated the effects of K9-C-peptide on high glucose-induced ROS generation and endothelial cell permeability in HRECs. High glucose concentrations induced intracellular ROS generation and TGase activation in HRECs (data not shown), which were inhibited by K9-C-peptide but not by K8 polypeptide **(Fig. 3A-C)**. Furthermore, K9-C-peptide, but not K8 polypeptide, attenuated *in vitro* endothelial permeability induced by high glucose conditions **(Fig. 3D)**. Consistent with a previous report 9, human C-peptide also inhibited ROS generation and microvascular permeability induced by high glucose conditions. These results demonstrate that the biological activity of K9-C-peptide is similar to that of human C-peptide in preventing high glucose-induced ROS generation, TGase activation, and vascular permeability in HRECs.

To support our *in vitro* findings, we investigated the inhibitory effects of K9-C-peptide on hyperglycemia-induced oxidative stress and vascular leakage in the retinas of NDPR mice. Compared with non-diabetic mice, NDPR mice displayed increased ROS generation and TGase activation in the retina, which was inhibited by a single intravitreal injection of K9-C-peptide **(Fig. 3E - H)**. Furthermore, NDPR mice displayed increased extravasation of FITC-dextran in the retina compared with non-diabetic mice, and this vascular leakage was attenuated by K9-C-peptide but not by K8 polypeptide **(Fig. 3I and J)**. Consistent with the pharmacokinetic results, a single injection of unconjugated human C-peptide inhibited oxidative stress, TGase activation, and microvascular leakage in the retinas of NPDR mice only for a single day, whereas a single injection of K9-C-peptide had much more prolonged effects.

### K9-C-peptide normalizes hyperglycemia-induced imbalance between VEGF and PEDF in the retinas of PDR mice

**Figure 4:**
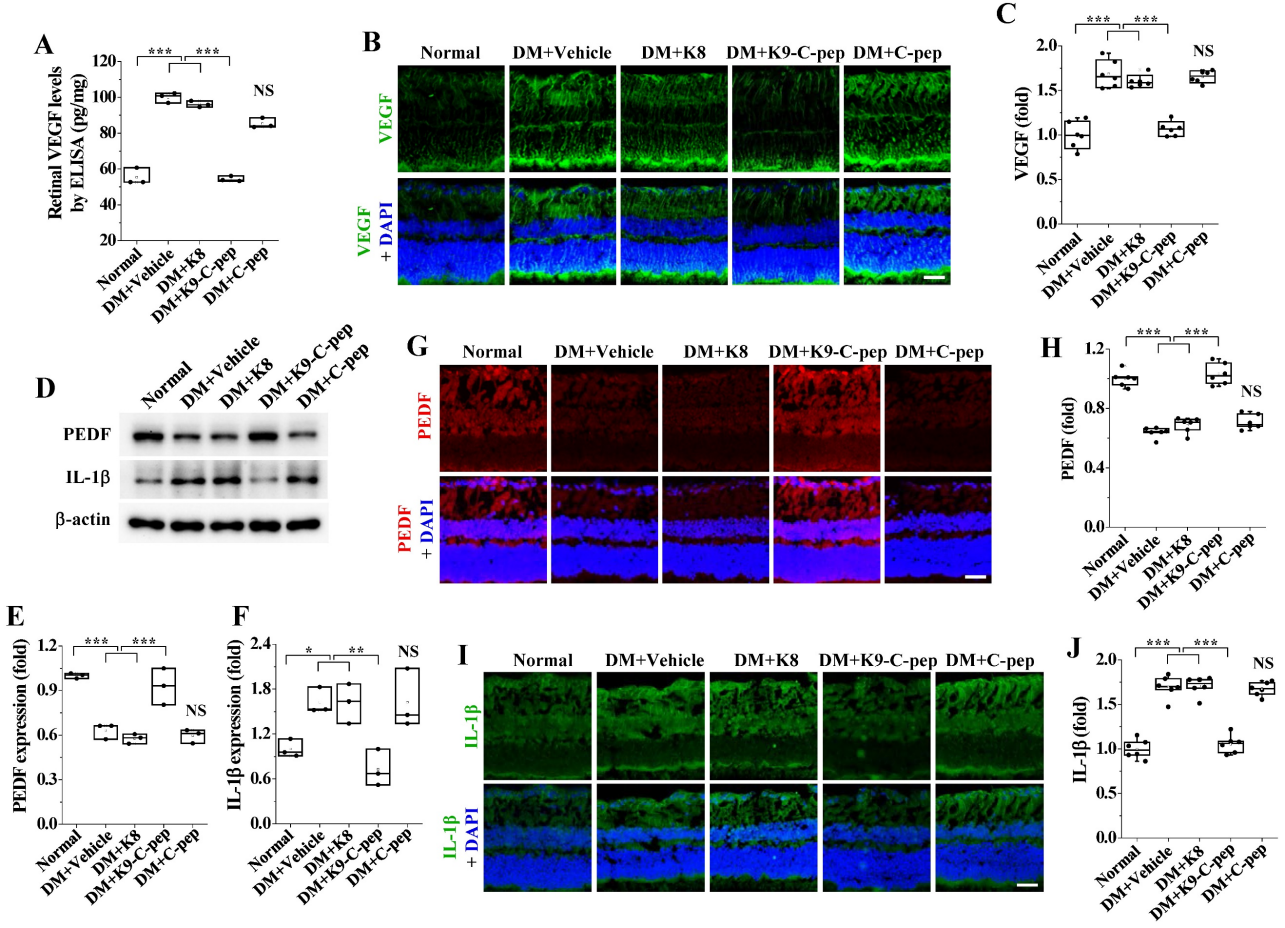
** Intravitreal K9-C-peptide inhibits VEGF overexpression, PEDF downregulation, and IL-1β expression in the retinas of PDR mice.** Six weeks after STZ injection, diabetic mice (DM) were supplemented for 16 weeks with two intravitreal injections of PBS (DM+Vehicle), K8 polypeptide (DM+K8), K9-C-peptide (DM+K9-C-pep), or C-peptide (DM+C-pep). The mice were then subjected to analysis of VEGF, PEDF, and IL-1β expression. **A-C.** VEGF expression in the retinas was analyzed by ELISA **(A)** and immunofluorescence **(B and C)**. **A.** Quantitation of retinal VEGF levels by ELISA (n = 3, two retinas/experiment). **B.** Representative images of VEGF expression (green) with nuclear counterstaining using DAPI (blue) in retinal sections. Scale bar, 100 μm. **C.** Quantitation of VEGF expression by measuring fluorescence intensity (n = 6). **D-J.** Expression of PEDF and IL-1β in the retinas was analyzed by Western blot **(D-F)** and immunofluorescence **(G-J)**. **D.** Representative Western blot images of PEDF and IL-1β expression in the retinas. **E and F.** Densitometric quantification of PEDF **(E)** and IL-1β **(F)** expression levels (n = 3, two retinas/experiment). **G and I.** Representative images of PEDF (red) and IL-1β (green) expression in retinal sections with nuclear counterstaining using DAPI (blue). Scale bar, 100 μm. **H and J.** Quantitation of PEDF and IL-1β expression by measuring fluorescence intensity (n = 6). Statistical significance was determined using one-way ANOVA with Holm-Sidak`s multiple comparisons test. NS, non-significant; ^∗^* P* < 0.05; ^∗∗^* P* < 0.01; ∗∗∗*P* < 0.001.

To understand how K9-C-peptide attenuates hyperglycemia-induced retinal angiogenesis, we investigated the effects of intravitreal K9-C-peptide on the hyperglycemia-induced imbalance of pro- and anti-angiogenic factors in the retinas of PDR mice. ELISA and immunofluorescence analysis indicated that hyperglycemia elevated the expression levels of the pro-angiogenic factor VEGF, and this overexpression was suppressed by K9-C-peptide (**Fig. 4A-C**). Conversely, Western blot and immunofluorescence analysis indicated that hyperglycemia reduced the expression levels of the anti-angiogenic factor PEDF, and this downregulation was reversed by K9-C-peptide **(Fig. 4D, E, G, and H)**. Neither K8 polypeptide nor unconjugated human C-peptide had any effect on hyperglycemia-induced VEGF overexpression or PEDF downregulation. These results demonstrate prolonged delivery of human C-peptide using K9-C-peptide normalized the hyperglycemia-induced imbalance between VEGF and PEDF in the retinas of PDR mice.

### K9-C-peptide attenuates hyperglycemia-induced inflammatory cytokine expression and microglial activation in the retinas of PDR mice

Inflammation contributes to the development of angiogenesis in PDR 27. Therefore, we investigated the inhibitory effect of intravitreal K9-C-peptide on microglia activation and expression of the inflammatory cytokine IL-1β in the retinas of PDR mice. Western blot and immunofluorescence assay demonstrated that IL-1β expression was elevated in the diabetic retinas, and the elevated IL-1β levels were attenuated by K9-C-peptide **(Fig. 4D, F, I, and J)**. In contrast, neither K8 polypeptide nor human C-peptide had any effect on the increased IL-1β expression in the retinas of PDR mice.

**Figure 5:**
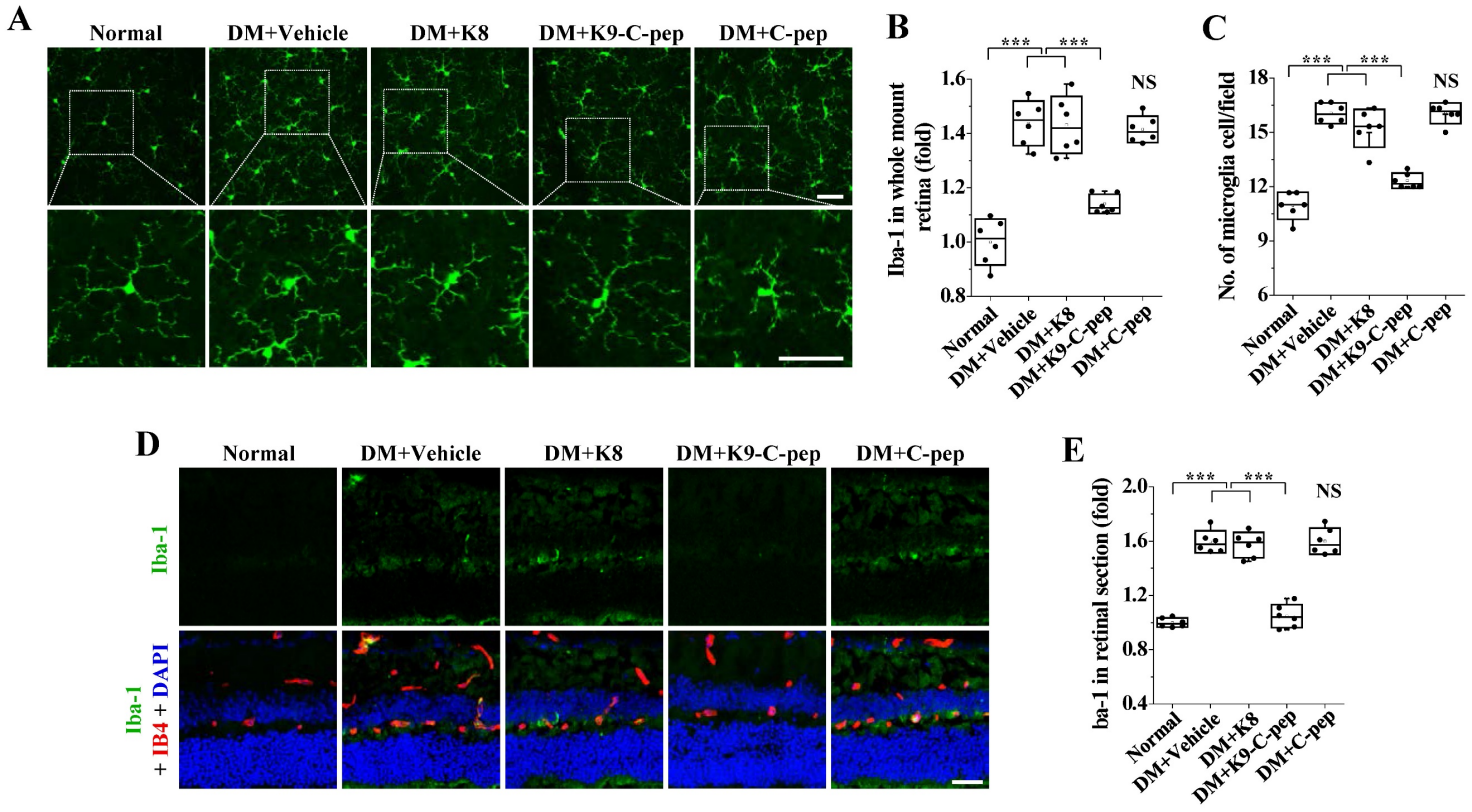
** Intravitreal K9-C-peptide inhibits hyperglycemia-induced microglial activation in the retinas of PDR mice.** Diabetic mice (DM) were supplemented for 16 weeks with two intravitreal injections of PBS (DM+Vehicle), K8 polypeptide (DM+K8), K9-C-peptide (DM+K9-C-pep), or C-peptide (DM+C-pep). Microglia activation was analyzed by immunofluorescence using anti-Iba-1 antibody in whole-mounted retinas **(A-C)** and retinal sections **(D and E)**.** A.** Representative images of microglia morphology and distribution in the deep plexus of whole-mounted retinas. The bottom row of each group displays enlarged images of the square areas in the top row. Scale bar, 50 μm. **B.** Quantitation of Iba-1 expression by measuring the fluorescence intensity in **A** (n = 6). **C.** Quantification of microglia per field in the deep plexus (n = 6). **D.** Representative images of Iba-1 expression (green) in retinal sections with nuclear counterstaining using DAPI (blue) and vessel staining using Alexa 647-isolectin B4 (red). Scale bar, 100 μm. **E.** Quantitation of Iba-1 expression by measuring the fluorescence intensity in **D** (n = 6). Statistical significance was determined using one-way ANOVA with Holm-Sidak`s multiple comparisons test. NS, non-significant; ∗∗∗*P* < 0.001.

We next examined the inhibitory effect of K9-C-peptide on microglial activation in the retinas of PDR mice. Compared with those of non-diabetic mice, the microglia of PDR mice had shorter and fewer branched processes in the deep plexus layer, which is indicative of microglia activation, and this sign of microglia activation was attenuated by K9-C-peptide **(Fig. 5A)**. Whole-mounted retinas and retinal sections of PDR mice displayed increased Iba-1 levels, and this increased Iba-1 expression was inhibited by K9-C-peptide **(Fig. 5A, B, D, and E)**. Furthermore, whole-mounted retinas of PDR mice showed increased numbers of microglia, which were normalized by K9-C-peptide **(Fig. 5A and C)**. Neither K8 polypeptide nor human C-peptide had any inhibitory effect on hyperglycemia-induced microglial activation. Taken together, these results indicate that K9-C-peptide had a prolonged, inhibitory effect on hyperglycemia-induced inflammatory responses in the retinas of PDR mice.

### Intravitreal K9-C-peptide attenuates BRB disruption in the retinas of PDR mice

**Figure 6:**
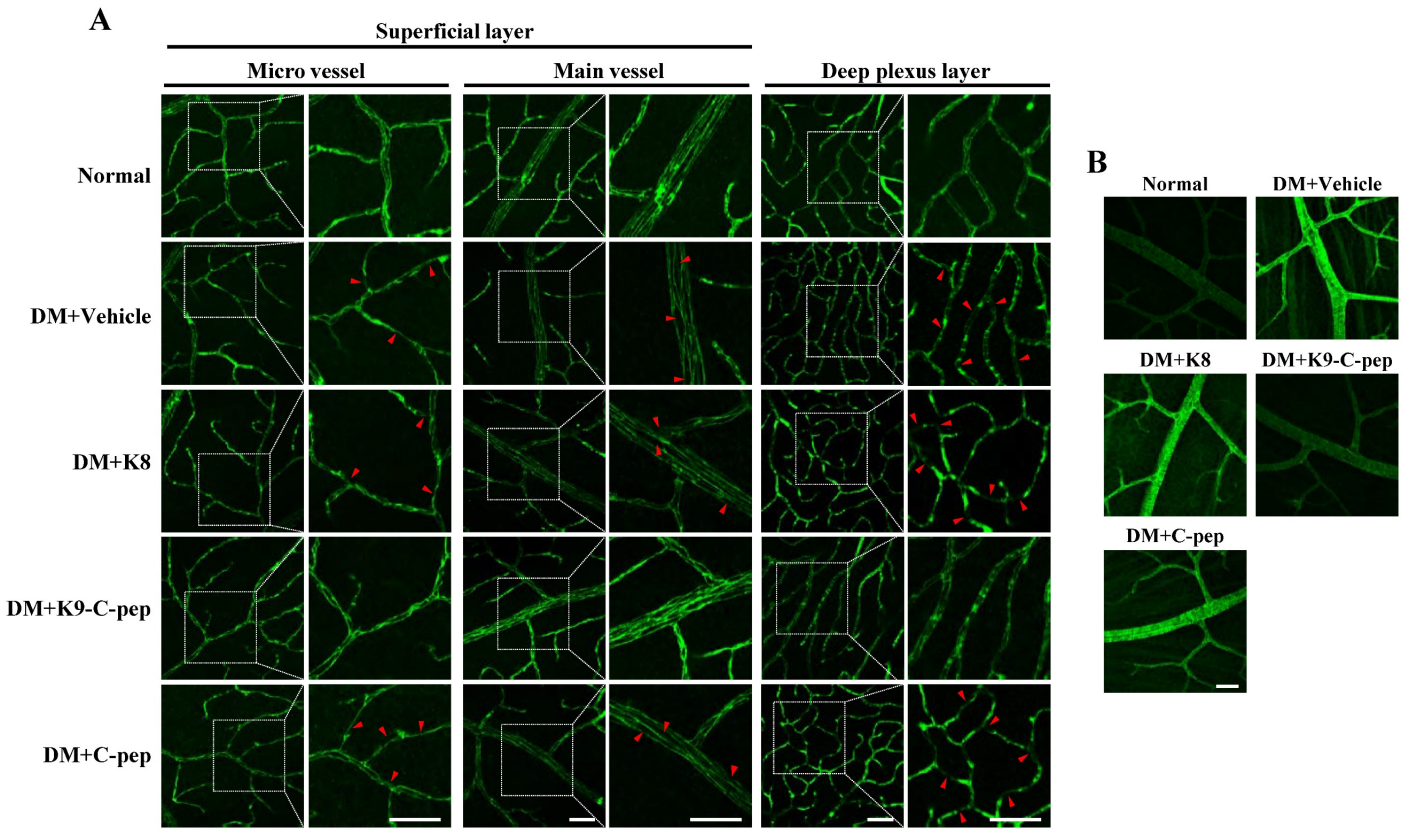
** Intravitreal K9-C-peptide inhibits hyperglycemia-induced VE-cadherin disassembly and stress fiber formation in the retinas of PDR mice.** Diabetic mice (DM) were supplemented for 16 weeks with two intravitreal injections of PBS (DM+Vehicle), K8 polypeptide (DM+K8), K9-C-peptide (DM+K9-C-pep), or C-peptide (DM+C-pep). VE-cadherin disruption **(A)** and stress fiber formation **(B)** were then analyzed in the retinas of the mice. **A.** Representative images of VE-cadherin in the superficial and deep plexus layers of whole-mounted retinas. The right column of each group displays enlarged images of the square areas in the left column. Arrows indicate disrupted adherens junction. Scale bar, 50 μm. **B.** Representative images of stress fibers in the deep plexus. Scale bar, 50 μm.

We next examined whether K9-C-peptide could inhibit adherens junction disassembly and stress fiber formation leading to BRB disruption in the retinas of PDR mice. Initially, we analyzed VE-cadherin within the microvessels and main vessels of the superficial and deep plexus layers. Compared with non-diabetic mice, PDR mice displayed downregulation and disassembly of VE-cadherin in both plexus layers, which was reversed by K9-C-peptide but not by K8 polypeptide or human C-peptide **(Fig. 6A)**. Hyperglycemia also enhanced stress fiber formation in the retinas of PDR mice, which was suppressed by K9-C-peptide **(Fig. 6B)**. These results indicated that K9-C-peptide ameliorates hyperglycemia-induced BRB disruption by inhibiting adherens junction disassembly and stress fiber formation in the retinas of DPR mice.

### Detailed investigation of pathological neovascularization in the deep plexus layer of PDR mouse retinas

**Figure 7:**
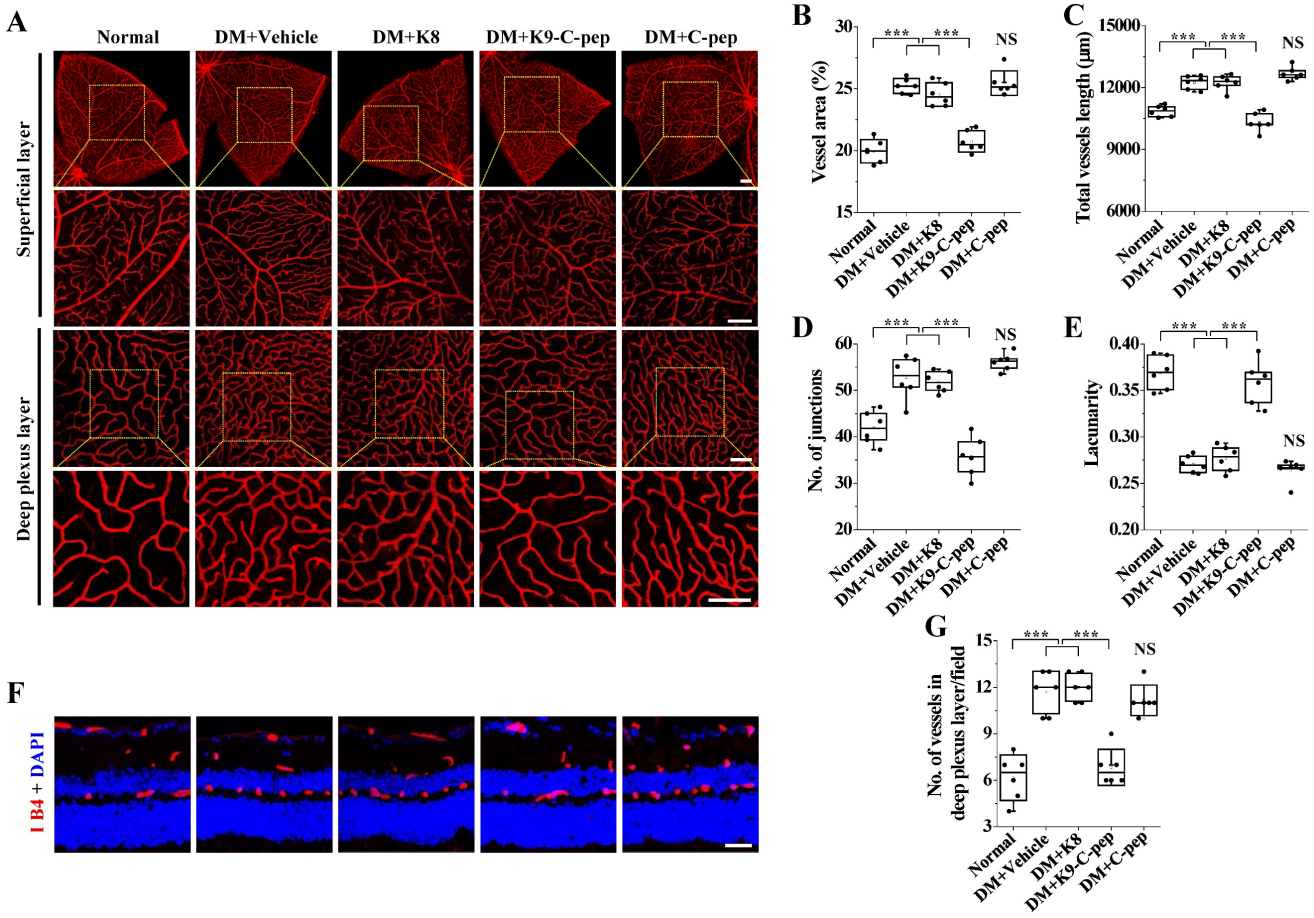
** Detailed investigation of pathological neovascularization in the deep plexus of PDR mouse retinas.** Diabetic mice were supplemented for 16 weeks with two intravitreal injections of PBS (DM+Vehicle), K8 polypeptide (DM+K8), K9-C-peptide (DM+K9-C-pep), or C-peptide (DM+C-pep). Angiogenesis was then visualized by Alexa 647-isolectin B4 staining (red) in whole-mounted retinas (**A-E**) and retinal sections (**F** and **G**). **A.** Representative images of vascular organization (red) in the superficial (Scale bar, 200 μm) and deep plexus (Scale bar, 100 μm) layers of whole-mounted retinas. The bottom row of each group displays enlarged images of the square areas in the top row. **B-E.** Quantitation of indexes of vascularity in whole-mounted retinas (n = 6): vascular area **(B)**, vessel length **(C)**, number of junctions **(D)**, and lacunarity **(E)**. **F.** Representative images of retinal vasculatures (red) in retinal sections with nuclear counterstaining using DAPI (blue). Scale bar, 100 μm. **G.** The number of vessels in the deep plexus layer of retinal sections (n = 6). Statistical significance was determined using one-way ANOVA with Holm-Sidak`s multiple comparisons test. NS, non-significant; ∗∗∗*P* < 0.001.

We analyzed the vascular organization in the superficial and deep plexus layers of whole-mounted retinas and retinal sections of PDR mice. Compared with those of non-diabetic mice, whole-mounted retinas of PDR mice displayed increased deep vascular density, as evidenced by decreased lacunarity and increased total vessel area, vessel length, and the number of junctions **(Fig. 7A-E)**. Critically, the changes in these parameters were normalized by K9-C-peptide, but not by K8 polypeptide or human C-peptide. In contrast to the deep vascular density, there was no significant increase in the superficial vascular density in the whole-mounted retinas of PDR mice. The finding of hyperglycemia-induced pathological neovascularization in the deep plexus was further supported by increased numbers of vessels in the deep plexus of retinal sections of PDR mice, which was alleviated by K9-C-peptide but not by K8 polypeptide or human C-peptide **(Fig. 7F and G)**. These results demonstrate intravitreal K9-C-peptide attenuated hyperglycemia-induced retinal neovascularization in the deep plexus of PDR mice.

## Discussion

**Figure 8:**
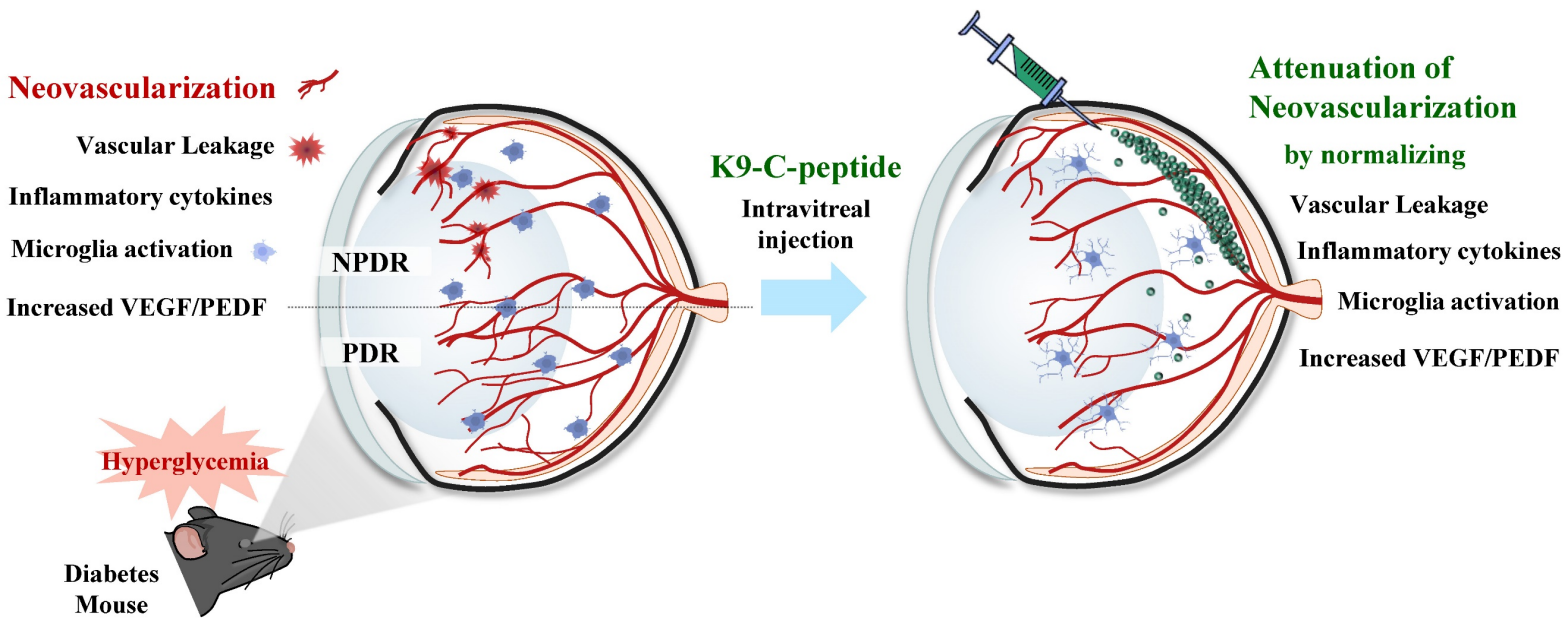
** Schematic depicting the ultra-prolonged therapeutic effect of intravitreal K9-C-peptide on vascular leakage, inflammation, microglia activation, and neovascularization in the retinas of diabetic mice.** NPDR, non-proliferative diabetic retinopathy; PDR, proliferative diabetic retinopathy; VEGF, vascular endothelial growth factor; PEDF, pigment epithelium-derived factor.

Pathological neovascularization, a hallmark of late-stage DR, causes major diabetic retinal disorders leading to vision loss 3. Most anti-DR drugs have short circulation half-lives, requiring repeated intraocular administration for sustained treatment 28. Therefore, a method for long-term drug delivery to treat patients with DR is needed. K9-C-peptide formed a depot in the vitreous chamber and slowly released human C-peptide into the intraocular space without inducing retinal cytotoxicity, suggesting that it may be suitable for prolonged treatment of diabetes-induced retinal neovascularization. Human C-peptide released from an intravitreal K9-C-peptide depot inhibited hyperglycemia-induced oxidative stress and vascular permeability in the retinas of NPDR mice. Intravitreal K9-C-peptide also provided prolonged attenuation of pathological events related to retinal neovascularization in PDR mice. Taken together, our results indicate that ultra-long-lasting delivery of human C-peptide from an intravitreal K9-C-peptide depot had a therapeutic effect against retinal neovascularization by normalizing hyperglycemia-induced oxidative stress, microvascular leakage, VEGF/PEDF imbalance, IL-1β expression, microglial activation, and BRB disruption **(Fig. 8)**.

Increased vascular permeability and angiogenesis are the main causes of vision loss in DR. Increased vascular leakage precedes pathological angiogenesis in DR, suggesting a close association between the two processes 29, which are both related to the interaction and balance of pro- and anti-angiogenic factors 30. Hyperglycemia increases the ratio of VEGF (a pro-angiogenic factor) to PEDF (an anti-angiogenic factor), which promotes diabetic retinal neovascularization. This disrupted balance between pro- and anti-angiogenic factors leads to oxidative stress, microvascular leakage, inflammation, BRB breakdown, and ultimately angiogenesis 31. In addition, several inflammatory cytokines and chemokines can act as angiogenesis inducers affecting endothelial cells; for instance, IL-1β and VEGF can upregulate each other and induce angiogenic responses 27, 32. Furthermore, there is accumulating evidence that microglia, the immune cells in the retina, can be activated by inflammatory conditions in DR, which can lead to retinal neurotoxicity, tissue damage, and angiogenesis 33, 34. With respect to BRB breakdown, VEGF signaling and angiogenesis are enhanced by disruption of VE-cadherin, that is important for maintaining the BRB and endothelial cell-contact integrity 35. In the present study, we demonstrated that intravitreal K9-C-peptide attenuates pathological retinal angiogenesis in PDR mice by normalizing oxidative stress, microvascular leakage, IL-1β expression, microglial activation, VEGF and PEDF balance, and BRB integrity, as determined by VE-cadherin disassembly and stress fiber formation. These results suggest that K9-C-peptide might be useful as an anti-angiogenic drug to treat DR.

Hyperglycemia-induced VEGF expression in the retina causes sequential increases in intracellular Ca^2+^ and ROS levels, which in turn lead to TGase2 activation and consequent stress fiber formation, VE-cadherin disassembly, and microvascular leakage 23, 24, 36. ROS generation and TGase2 activation were shown to be in a vicious cycle in the aortic endothelium of diabetic mice and this vicious cycle plays an important role in sustained expression of inflammatory adhesion molecules and apoptosis 22. ROS-mediated TGase2 activation also plays a role in hyperglycemia-induced vascular leakage in the glomeruli and lungs of diabetic mice 11, 26. We previously showed that human C-peptide attenuates hyperglycemia-induced microvascular leakage in the retina by inhibiting VEGF-induced ROS generation and TGase2 activation 9, 24. In a separate study, hyperglycemia-induced expression of Iba-1, a biomarker of microglial cell activation, was inhibited by human C-peptide or the antioxidant Trolox 25. Consistent with the previous results, in the present study, intravitreal K9-C-peptide inhibited ROS generation and TGase activation, which was mostly contributed by TGase2 24, and subsequent microvascular leakage in the retinas of diabetic mice and HRECs. Moreover, K9-C-peptide normalized hyperglycemia-induced stress fiber formation and VE-cadherin disassembly, leading to BRB breakdown, inflammatory cytokine expression, and microglia activation. These results suggest that ROS generation and TGase2 activation play a key role in the mechanism by which K9-C-peptide attenuates the hyperglycemia-induced pathological events associated with retinal neovascularization.

Several therapeutic strategies for topical, systemic, periocular, or intraocular delivery to the eyes of patients with DR have been reported 14, 28. However, each of these strategies has adverse effects and limitations. Topical administration, where drugs are delivered through the anterior segment of the eye, may result in limited drug bioavailability due to the unique anatomy of the eye 14, 37. Systemic delivery by oral or intravenous routes has the disadvantages of enhanced drug clearance due to active transport systems and limited distribution to the eye because of the BRB 28. Periocular delivery is less invasive than intraocular delivery, but it also has the disadvantage of limited drug bioavailability because of the BRB 38. Intravitreal injection, the typical drug delivery route for DR therapy, is the most direct and efficient way to deliver drugs to the retina 14; however, it must be repeated frequently, which can cause side effects such as anxiety, pain, infection, and tissue damage 15. To reduce the inconvenience and side effects caused by repeated injections, systems for sustained drug delivery to the retina have been examined, including intravitreal implants, microparticles, nanoparticles, and liposomes 13. These approaches have their own limitations, including elevated cataract incidence, limited biocompatibility and biodegradability, and aggregation 39-41. K9-C-peptide may provide a means to overcome these limitations and enable long-term delivery of human C-peptide to ameliorate hyperglycemia-induced neovascularization in PDR.

ELPs have been actively targeted for controlled drug delivery due to their temperature-sensitive behavior with a transition temperature, which is dependent on the biopolymer concentration. These biopolymers have additional advantages including biodegradability, non-toxicity, and high biocompatibility in the body 19. We previously developed K9-C-peptide, an ELP-conjugated form of human C-peptide that has reversible thermal-phase behavior and is degraded by proteases, including elastase-2 and collagenase-2 20. Subcutaneously injected K9-C-peptide formed a hydrogel depot that slowly released human C-peptide into the blood circulation for 19 days 21. In the present study, we found that an intravitreal K9-C-peptide depot slowly released human C-peptide and maintained physiological levels of C-peptide in the intraocular space of mice for at least 56 days. This sustained delivery of human C-peptide attenuated neovascularization through inhibiting hyperglycemia-induced pathological events in the retinas of PDR mice. Furthermore, K9-C-peptide did not cause any detectable inflammation, apoptosis, or pathological changes in the retinas of the mice. Thus, our results suggest intravitreal K9-C-peptide has good bioavailability and biocompatibility for long-term delivery of human C-peptide and thus may provide a patient-friendly therapy for DR by overcoming the clinical disadvantages of current anti-DR drugs, including short circulation half-lives and the need for frequent intraocular administration.

In conclusion, intravitreal K9-C-peptide ameliorated hyperglycemia-induced retinal neovascularization in diabetic mice. An intravitreal K9-C-peptide depot slowly released human C-peptide for at least 56 days into the intraocular space. This ultra-long-lasting human C-peptide delivery attenuated pathological events related to retinal neovascularization in PDR mice, including oxidative stress and vascular leakage, imbalance between pro- and anti-angiogenic factors, inflammatory responses, and BRB breakdown. In contrast, unconjugated human C-peptide did not have these effects. Thus, K9-C-peptide may be useful for prolonged intraocular delivery of human C-peptide as an anti-angiogenic agent for long-lasting treatment of DR.

## Abbreviations

BRB: blood-retinal barrier
DME: diabetic macular edema
DR: diabetic retinopathy
ELP: elastin-like polypeptides
H_2_DCFDA: dichlorodihydrofluorescein diacetate
Iba-1: ionized calcium-binding adaptor molecule 1
ITC: inverse transition cycling
NPDR: non-proliferative diabetic retinopathy
PDR: proliferative diabetic retinopathy
PEDF: pigment epithelium-derived factor
PEG: polyethylene glycol
ROS: reactive oxygen species
VEGF: vascular endothelial growth factor
